# Supplementary figures and images for: Zyxin contributes to coupling between cell junctions and contractile actomyosin networks during apical constriction
Source: PLoS Genet. 2023 Mar 28;19(3):e1010319. doi: 10.1371/journal.pgen.1010319 (PMC10081768; doi:10.1371/journal.pgen.1010319)

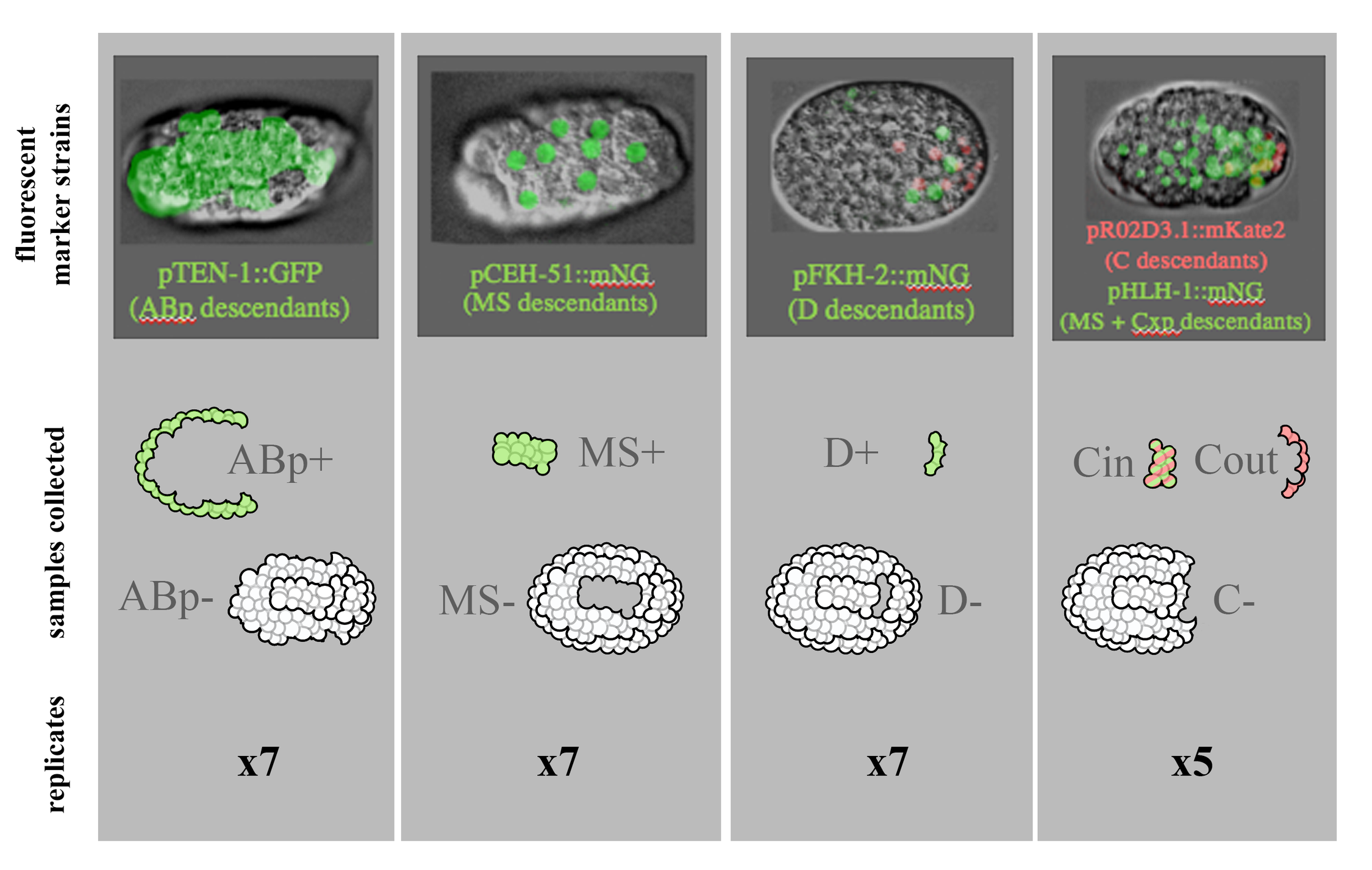

Supplement: S1 Fig — Four fluorescent marker strains that were used to dissect and collect each sample collected from the 100 cell stage. (TIF) [file pgen.1010319.s001.tif]

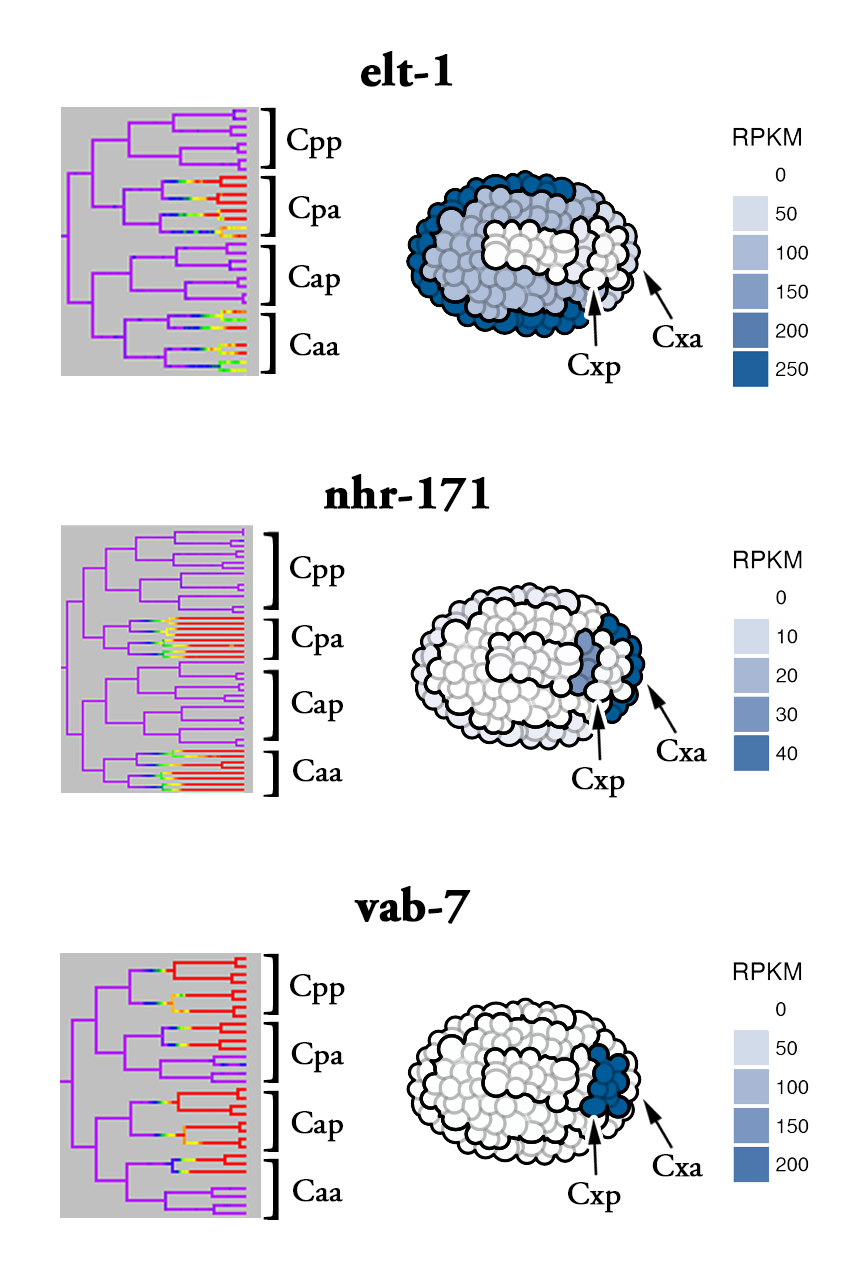

Supplement: S2 Fig — Three genes with protein expression in C descendants were selected from the Waterston Lab’s lineagomics database (epic.gs.washington.edu) and compared to our transcriptome data. Cell lineages show only the C descendants, and are color coded by relative fluorescence levels detected from a film of embryonic development taken of embryos with multi-copy arrays of promoter fusions of each gene. According to the Waterston lineages (left), transcripts of elt-1 and nhr-171 are expected to be enriched in Cxa descendants, and transcripts of vab-7 are expected to be enriched in descendants of Cxp, which we also see in our transcriptomes (right). (TIF) [file pgen.1010319.s002.tif]

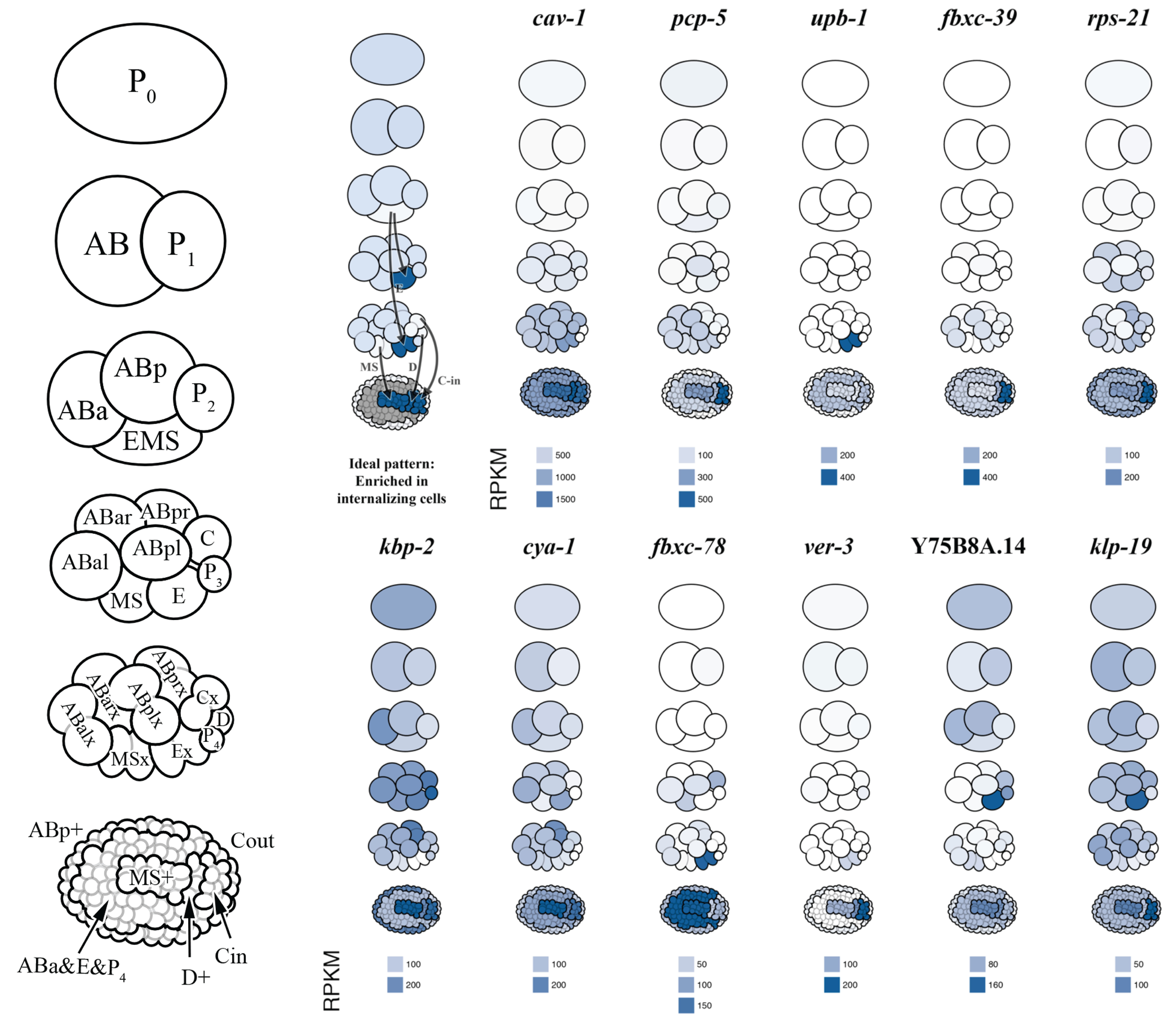

Supplement: S3 Fig — Pictograms showing individual cell heat map expression patterns of the genes indicated with RPKM values listed. None of these 11 candidates had expression patterns that perfectly matched with cell internalization, i.e. enrichment was weak in one or more groups of internalizing cells, or some non-internalizing cells showed strong expression. (TIF) [file pgen.1010319.s003.tif]

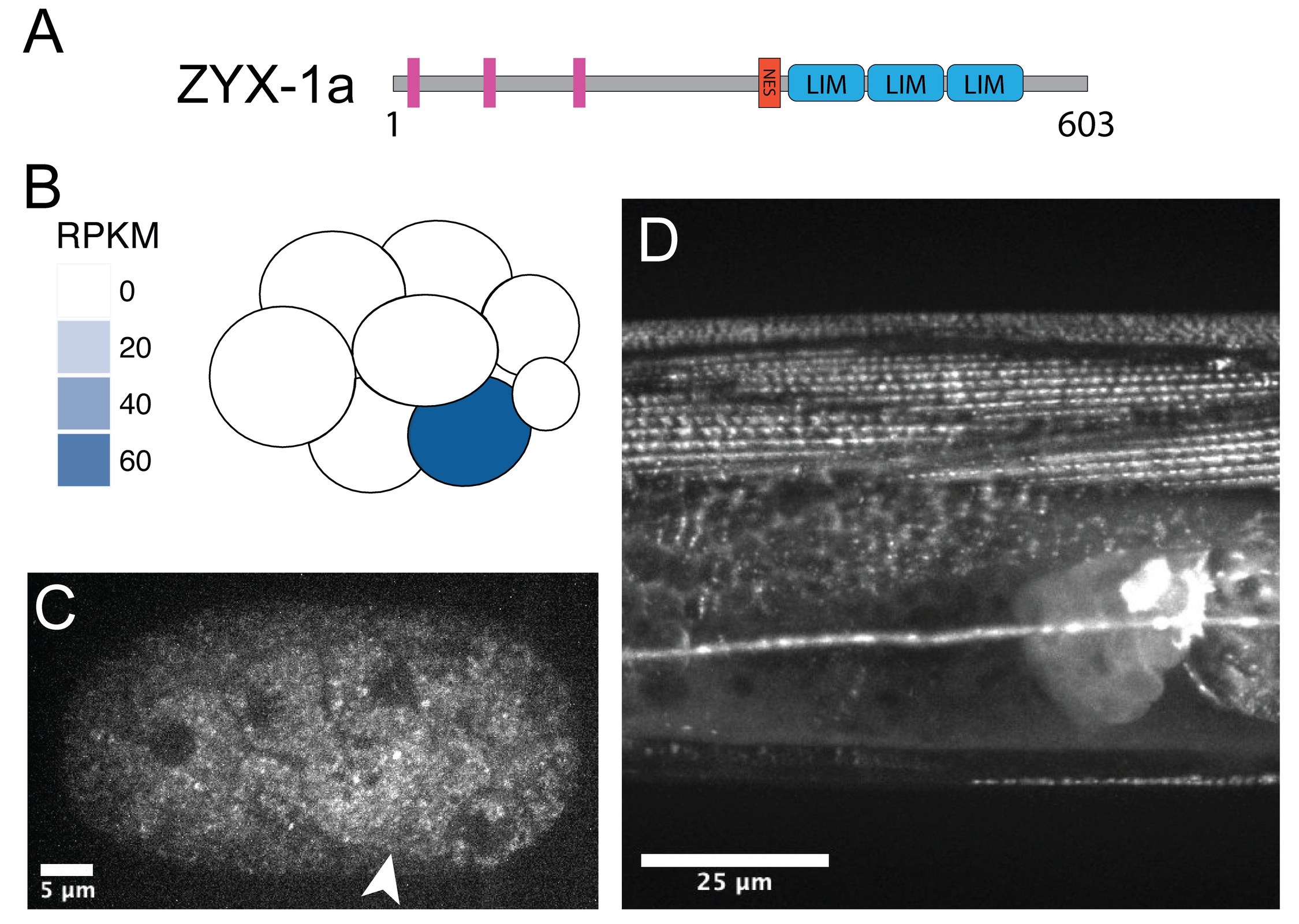

Supplement: S4 Fig — (A) C. elegans ZYX-1a consists of a few proline rich regions near the N-terminus (magenta boxes), a predicted nuclear export sequence (NES, orange box), and 3 tandem LIM domains. (B) zyx-1 is expressed at the 8 cell stage in EPCs (19). Pictogram key in S4 Fig. (C) Endogenous mNG-tagged ZYX-1 does not accumulate in EPCs (white arrowhead) appreciably above background, although the expression levels (B) are predicted to be low at this stage. This image was taken with settings that amplified even low level fluorescence and background in an attempt to enhance any apparent signal. We also failed to detect ZYX-1 by immunostaining ZYX-1::GFP embryos and using TSA amplification. (D) Endogenous mNG-tagged ZYX-1 shows clear expression in young adults, with localization patterns matching previous reports for zyx-1 transgenes (46), suggesting that our tagged gene is properly expressed. (TIF) [file pgen.1010319.s004.tif]

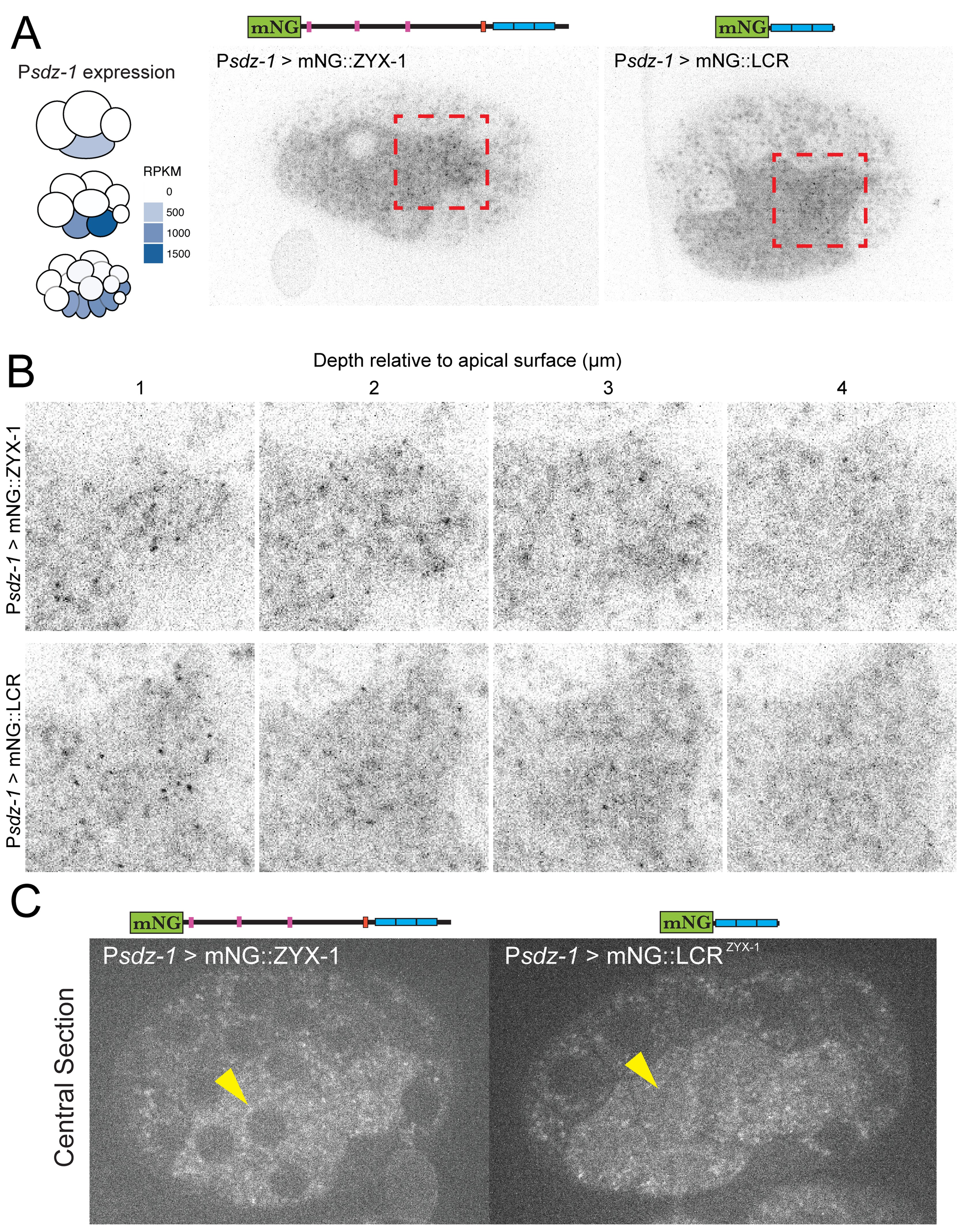

Supplement: S5 Fig — (A) An mNG-tagged transgene driven by the sdz-1 promoter, which drives expression in MS and E cell lineages ~20-fold higher than predicted levels for endogenous zyx-1 (left). (B) mNG-containing puncta can be seen at the apical surface for both full length zyxin and the LIM domain-containing region (LCR) of ZYX-1. Apical slices at the depths indicated show more puncta closer to the apical surface, with fewer punta appearing further away from the surface. (C) The predicted NES is functional. If viewed from a central section, the LCRZYX-1 construct, which lacks the NES, is not excluded from the nucleus (yellow arrowheads). (TIF) [file pgen.1010319.s005.tif]

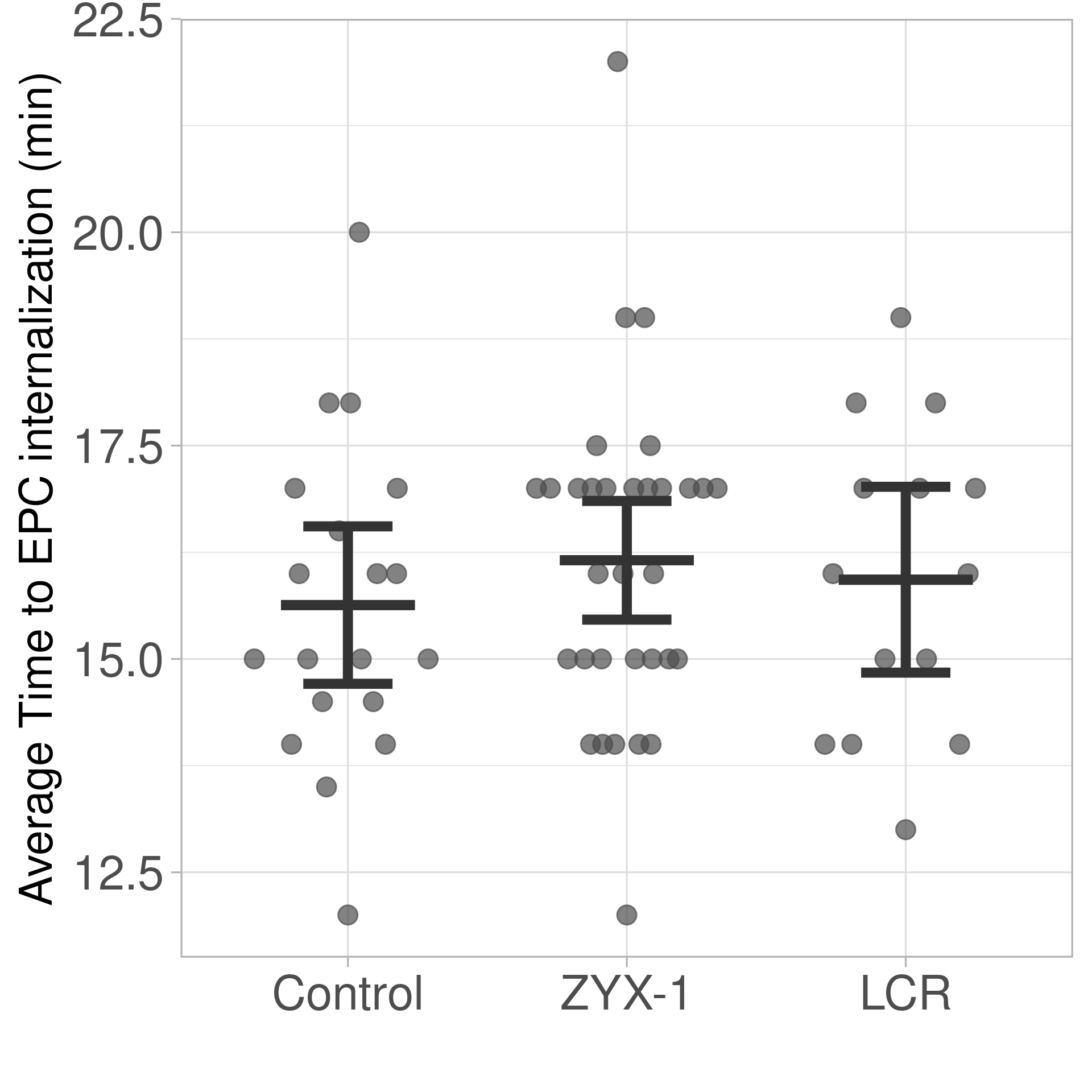

Supplement: S6 Fig — Plot of the time it took between MSxx birth and EPC internalization, in minutes. Psdz-1 driven overexpression of neither mNG::ZYX-1 nor mNG::LCRZYX-1 affected the timing of cell internalization as compared to control embryos (ZYX-1, p = 0.34, n = 32; LCRZYX-1, p = 0.65, n = 14). (TIF) [file pgen.1010319.s006.tif]

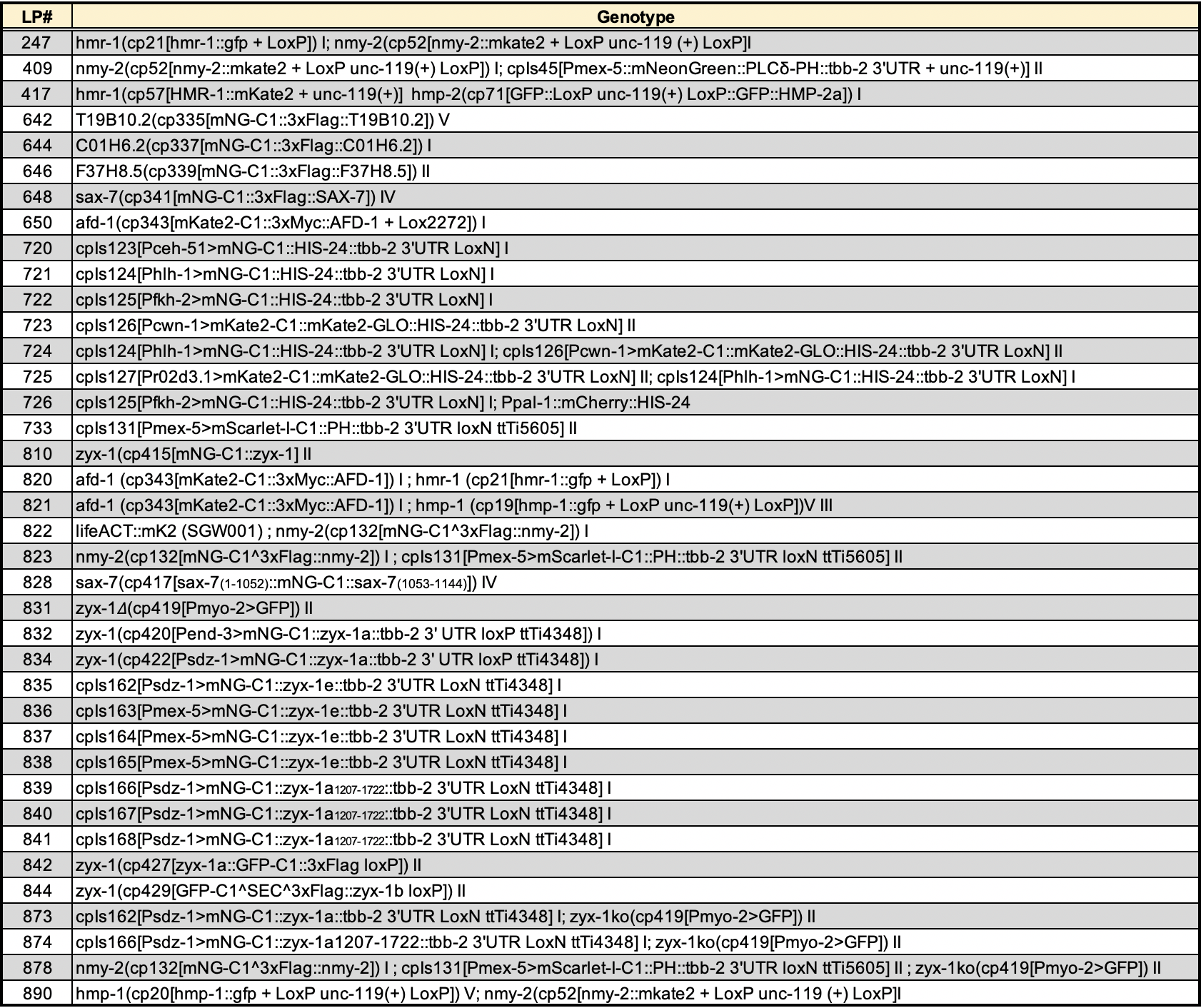

Supplement: S1 Table — (TIF) [file pgen.1010319.s007.tif]

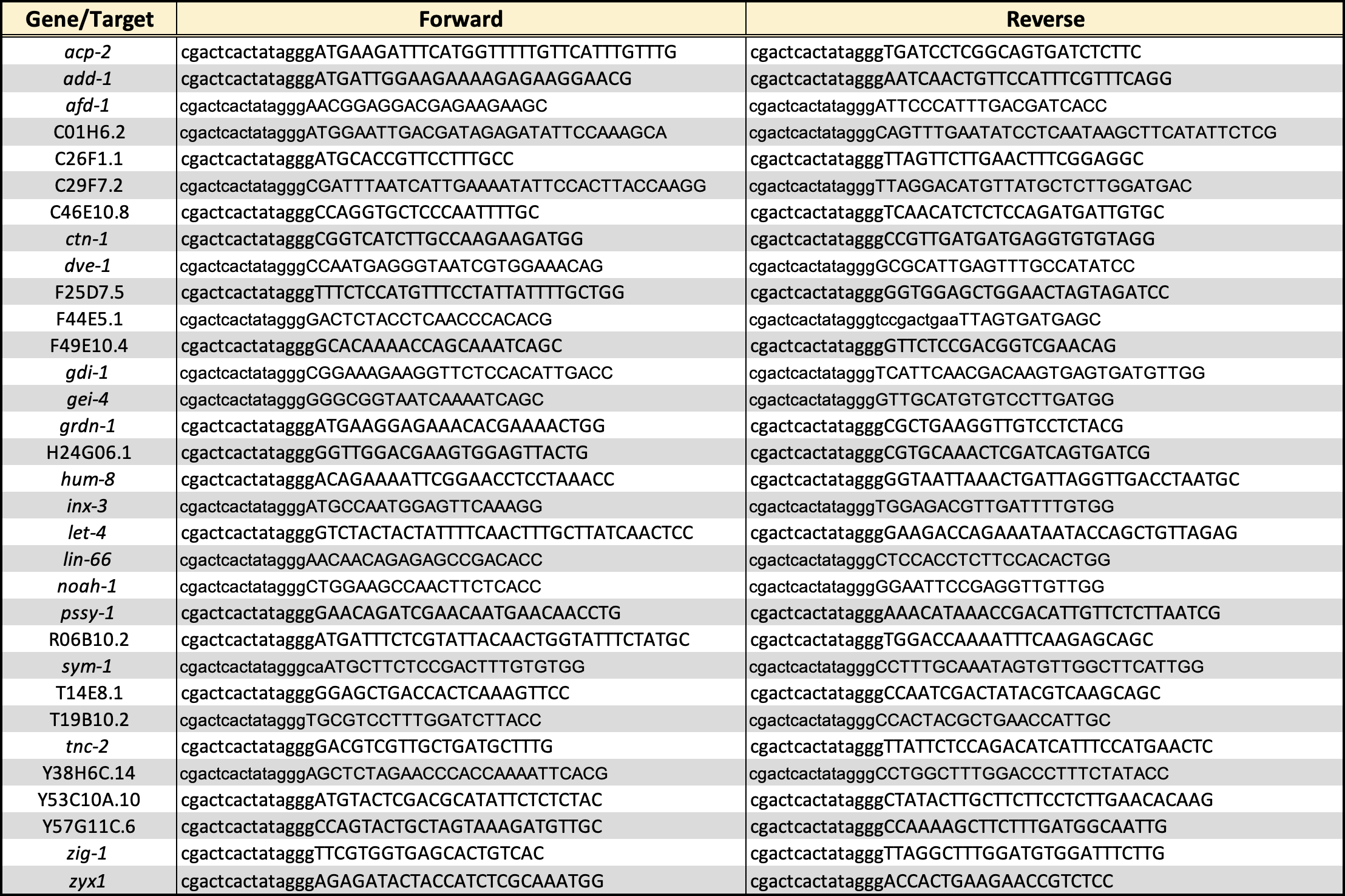

Supplement: S2 Table — (TIF) [file pgen.1010319.s008.tif]

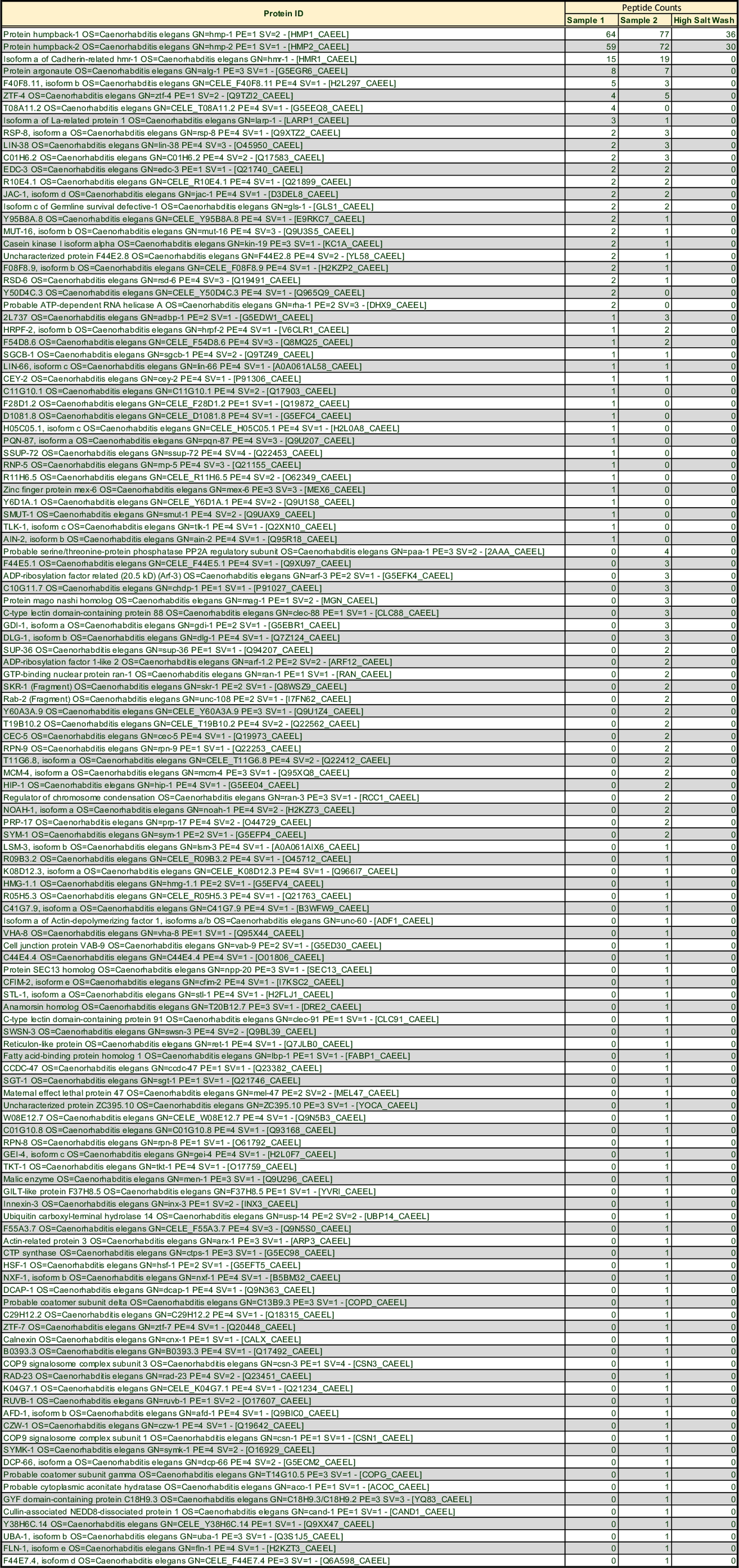

Supplement: S3 Table — (TIF) [file pgen.1010319.s009.tif]
