# Supplementary material for: Zyxin contributes to coupling between cell junctions and contractile actomyosin networks during apical constriction
Source: PLoS Genet. 2023 Mar 28;19(3):e1010319. doi: 10.1371/journal.pgen.1010319 (PMC10081768; doi:10.1371/journal.pgen.1010319)
Supplement: Attachment — Submitted filename: Resubmission Response to Reviewers.pdf [file pgen.1010319.s012.pdf]

Response to reviewers

Based on the comments from all three reviewers, we have added new experiments and buttressed previous ones with additional data (new data are presented in Fig 2D, 2F, 3E, 6A-C, Table 2, and Supplementary Fig 5B). This has resulted in a significant focusing of the work's contributions on two central points: the work identifies several new genes that contribute to *C. elegans* gastrulation, and it pinpoints a role for ZYX-1/zyxin as a long-sought player that contributes directly or indirectly to linking junctions to contracting actomyosin networks (Abstract, p. 3). We make clearer now that screening for key players was necessarily far more labor-intensive than in typical screens, requiring "integrating diverse methodologies including generating new image analysis resources, as well as screening for defects in a complex process involving cellular and subcellular dynamics" (p. 6). We are thrilled that the significant effort paid off, pinpointing a key role for zyxin, which is expressed at just the right time and in the right cells (Fig 4C), and without which junctions are not pulled in nearly as efficiently as in normal embryos (Figure 6B,C). We have removed a focus on afadin in limiting slippage based on our additional data; as reviewers pointed out, afadin has known roles in connecting membranes to actin networks, but our data (additional analysis added as Fig 6B,C and Table 2) do not implicate it in the specific form of temporally-regulated linkage that is the heart of what we sought in this work. We're grateful to reviewers for making suggestions that sharpened the focus of the paper and also that prompted us to articulate more clearly some key points.

Reviewer #1: Slobodnick et al describe their screen for factors that contribute to gastrulation and identify AFD-1 and ZXY-1 as factors contribute to gastrulation, in an effort to identify factors that link myosin contraction to the cadherin junctions and membrane. They also show that AFD-1 is cadherin-dependently enriched in Ea/Ep border, where cadherin localizes, with apical enrichment. The authors also develop and use a semi-automated image analysis pipeline to show that knockdown of either AFD-1 or ZXY-1 leads to a loss in the difference in "slippage" from early to late time points in the internalization/gastrulation process. These results are interesting and do implicate AFD-1 and ZXY-1 in gastrulation and slippage. However, as written the manuscript is somewhat incremental in advancing our understanding of these factors during gastrulation, and the authors also should provide a more in depth analysis of some of their data to better compared the mutant phenotypes to the control, as detailed below. As the authors point out in the introduction, Afadin already has been implicated in linking membrane proteins to actin, and the expression data on zyxin is based on over-expression. Without a more extensive analysis, the manuscript as written does not in this reviewer's opinion provide an sufficient advance in our understanding of gastrulation for publication in PLOS Genetics.

We have conducted new experiments and provided additional data on previous experiments (including increasing n values). These efforts have resulted in a significant focusing of the work's contributions on two central points: the work identifies several new genes that contribute to *C. elegans* gastrulation, and it pinpoints a role for

ZYX-1/zyxin as a long-sought player that contributes directly or indirectly to the temporally-regulated linking of junctions to contracting actomyosin networks (Abstract, p. 3). We articulate more clearly now the originality of this second contribution: "Although many proteins have been identified at sites of cadherin-based adhesion that could feasibly serve as such temporally regulated links, the specific, temporally-regulated links relevant to triggering apical constriction are not yet known in any system" (Introduction, p. 6). Zyxin is expressed at just the right time and in the right cells (Fig 4C), and without it, junctions are not pulled in nearly as efficiently as in normal embryos (Figure 6B,C). In line with PLoS Genetics' criteria for publication, we believe that this supports that the work is important and of broad interest because it points to a "way that developmental patterning spatiotemporally regulates cell biological mechanisms *in vivo*" (Abstract, p. 3).

Major comments.

1. The authors should explain how they paint with green pseudo color in Fig2B. Without an explanation, the figure is uninterpretable; why is the mutant cell a darker green and how did they do the pseudocoloring to indicate this? Figure 2A clearly shows the gastrulation defect , and thus Fig2B is not really needed but if included should be described in more detail.
We agree that Fig 2B is not essential, so we have removed it as the reviewer suggested.

2. The authors use over-expression to assess ZYX-1 localization with an mNeonGreen fusion. Given the over-expression caveat, an antibody that can detect endogenous ZYX-1 would be more convincing if such antibodies exist. At a minimum, the authors should show multiple focal planes if they want to assert that the detected over-expressed fusion is apically enriched; the supplemental figure panel only shows an apical section and no other sections, and thus one cannot assess any apical enrichment.
It is possible that ZYX-1 functions specifically at very low levels just as the protein is first made, making it inherently difficult to visualize. We discuss this now on p. 18, lines 4-5. At the reviewer's suggestion, we tried using immunofluorescence – using antibodies against GFP to amplify signal for ZYX-1::GFP in fixed embryos, and we have tried TSA amplification to try to boost signal. Unfortunately, neither worked (Supp Fig 4 legend). In line with the reviewer's comments, we have now added multiple focal planes of Psdz-1>mNG::ZYX-1 and for ZYX-1's LCR (Supp Fig 5B) so that readers can see the cortical enrichment. We also found that using this driver to express ZYX-1 only in E cells and their cousin MS cells (using Psdz-1>mNG::ZYX-1) can rescue gastrulation defects, whereas expressing only a LIM domain-containing part of ZYX-1 similarly cannot (p. 19). This buttresses our finding that despite being expressed at a low level – detectable to date only in mRNA data (Fig 4B) but not protein localization data during gastrulation – ZYX-1 is playing an important role in gastrulation. We are excited about the prospect that it may function specifically at very low levels, just as expression begins; we now propose alternative mechanisms by which it may act to promote dynamic coupling of membranes to contracting actomyosin networks: "We speculate that ZYX-1 might normally localize to junctions and indirectly strengthen mechanical connections between junctions and

contracting actomyosin networks. Alternatively, ZYX-1 might function throughout the apical actin cortex and contribute to coupling by enabling rapid repair of stressed actin filaments."

3. The authors never analyze gastrulation defects in a afd-1, zyx-1 double mutant, based on Nomarski images like those shown in Figure 2. In Figure 6, the authors show no significant difference between this double mutant and the single mutants with respect to myosin velocity or slippage. This is somewhat surprising and even suggests that they may act in the same pathway, as opposed to parallel pathways or contributions. The authors should assess the GAD phenotype as scored by Nomarski over time in the double mutants vs single mutants to verify that there is no additive or synergistic interaction.
We regret this oversight; we have now added data on the double, which did not increase penetrance (p. 17). We did not interpret this as meaning that the two proteins act in the same pathway because the double *null* allele background required to make this conclusion with confidence would not be viable.

4. The authors base their conclusion (that AFD-1 and ZYX-1 contribute to the linkage between myosin and the apical membrane/cadherin complex) on the loss of any difference in slippage between early and late time points during gastrulation. While this is an interesting observation, it is not clear to this reviewer how this implicates these proteins in this process, and the analysis could be much more extensive with respect to the comparisons of both early and late time points in the mutants, to the early and late time points in the control embryos. The authors compare early vs early slippage in each mutant vs control in Table 2 but do not provide the same analysis for late slippage. Indeed, inspection of Figure 6B and Table 2 indicates that the slippage rate may actually decrease in the double mutant relative to the control and single mutants (if the authors would examine more embryos); such a result would not be predicted for proteins that promote linkage between the apical membrane/junction and myosin. If anything the slippage rate should increase, and it appears it may decrease, as striking the p value for early mutant vs early control is much smaller for the double mutant compared to each single mutant. Moreover, the number of embryos analyzed is small (only 6, 6, 7 and 9). While the data points per embryo are large, embryo to embryo variability is a common phenomenon at the cell biological level in early embryos, and the authors need to increase the number of embryos analyzed to more carefully and conclusively assess any impact of the double mutant on early and late slippage rates.
We agree with the reviewer that more analysis and higher n values would be ideal for the reasons raised, so we collected more data. We thank the reviewer for urging us to do so. We now report data from thousands of measurements (dots in Fig 6C) and for a greater number of embryos: 10 control embryos, 9 *afd-1* RNAi, 10 *zyx-1* knockout, and 12 double *afd-1* RNAi and *zyx-1* knockout. We also present more thorough comparisons of myosin velocity, rate of membrane movement, and slippage rate so that readers can assess each of these along with the relevant statistics (Table 2, page 23). To summarize the data, we had predicted that (1) loss of a long-sought gene required for actomyosin-junction coupling would have little to no effect on actomyosin contraction rate (although subtle effects are possible given afadin and zyxin's known roles, we confirmed that the changes

we see in the rate of myosin's inward movement were not statistically significant in Fig 6A; Table 2 on p. 23). We also predicted that (2) the expected reduction of actomyosin-membrane slippage over time that occurs in normal embryos (i.e., engaging the hypothesized clutch – mechanically linking membranes to actomyosin) would be mitigated or lost altogether under loss of function of genes of the type that we sought. This prediction, that was confirmed by our previous data, also holds true with the additional data for *zyx-1* (Fig 6C). Our additional data do not support strongly that *afd-1* affects late membrane movement rates or slippage (Fig 6B; Table 2 on p. 23). Hence the manuscript leaves *afd-1* as among genes identified with roles in gastrulation, but *zyx-1* is the focus of the manuscript because it alone has a clear role in reducing slippage over time.

So that readers can assess all of the data more fully, we now also present the inward membrane rates in a new Fig 6B.

We expect that our writing and additional data presented in response to the reviewer's comments here will be helpful for readers to think clearly about the results. Given that the normal loss of slippage over time (Fig 6C control, and Table 2) is reduced significantly in the absence of zyxin (Fig 6C zyx-1Δ, and Table 2), we think our conclusion that these genes contribute to the reduction in slippage is a logical conclusion. We thank the reviewer for their helpful comments.

Minor comments.

1. In Figure 1, the authors show that cadherin and beta-catenin co-localize and stay co-localized, whereas myosin and alpha-catenin start out co-localized but then diverge, with myosin moving in ahead of alpha-catenin. However, in the text the authors say that Figure 1 shows that both alpha and beta-catenin co-localize with cadherin, and that both lag behind myosin during constriction. The figures only shows beta-catenin co-localizing with cadherin (not alpha-catenin) and only shows alpha-catenin trailing myosin (and not beta-catenin). The authors either need to add more data to Figure 1 or revise the text to accurately describe the more limited figure.
We have adjusted the wording to be more carefully in line with what we are showing in Fig 1, "mKate2-tagged HMR-1/cadherin localized at apical cell-cell boundaries, as did GFP::HMP-2/β-catenin and HMP-1/α-catenin::GFP" (p. 8).

2. The text at the top of page 15 (rest of a paragraph) could mostly be moved to the methods.
We have now streamlined this description of filtering to pinpoint genes of interest.

3. Should the authors refer to the "internalization" of the analyzed cells, rather than to "gastrulation" given that the cells undergoing internalization are not endodermal?
Because all germ layers except ectoderm gastrulate in most animals (i.e. endoderm, mesoderm, and germline), we have kept the language unchanged in the resubmission.

4. The authors use the term “master” regulator. Like chromosome “segregation” during mitosis, these terms (even more so, “master” regulator) reek of structural racism and should be disposed of in the scientific literature. The concept of a master regulator in particular has been absurd for decades now, given the complexity of biology. The use of the term seems more geared to promoting the reputation of investigators than to advancing our understanding of biology in any meaningful way. Proteins are required or not. Let’s drop the sensationalism. And especially please stop using that offensive and absurdly ego-oriented term, master-regulator. It makes me not want to read this paper again.
We are grateful to the reviewer for raising this point. We have removed this term (and we have used this learning experience to spark some reading and useful discussion in the lab that will help us avoid this error in the future). We now use alternative language, “snail family genes that orchestrate another cell shape change”. We chose this wording carefully to retain concisely a hint of snail family genes’ necessity and sufficiency to cause a complex cell shape change, in diverse cells, akin to what we screened for taking advantage of the multiple cells that gastrulate by apical constriction in *C. elegans*.

Reviewer #2: Slabodnick et al describe two screens (proteome and transcriptome) for regulators of C. elegans gastrulation, and characterize the function of two of the hits, namely afadin and zyxin, to couple the contracting apical actomyosin to cell junctions to exert the pulling force. While C. elegans is renowned as a genetic system, the gastrulation process has long defied genetic screens. This study continues a long-term and admirable effort of the Goldstein lab to push the screens with cutting edge techniques, and the results are valuable to the field. The fast live imaging and the semi-automatic image analysis provide solid support for the function of afadin and zyxin to couple actomyosin and cell junctions. My comments are on minor details of the manuscript.

1. Table 1 names the developmental stages as 8- and 16-cell, while the main text as 8- and 24-cell. Please reconcile.
We corrected all of these to 24-cell and added a brief explanation (Methods p. 25).

2. Fig 1B. It appears that the conclusion comes from the kymograph, and the line scan does not seem to contribute. If I missed the point, please clarify. Otherwise, consider removing or de-emphasizing the line scan panels.
We have kept this figure in because the line scans make clear that the bulk of the alpha-catenin and beta-catenin remains at cell-cell boundaries. We had neglected to refer to Fig. 1 parts (A and B) separately in the text to help readers appreciate this point. We have done so now (p. 8).

3. Localization of afadin. Fig 2E-F focus on the junction of Ea/p. The more interesting junctions would be those of Ea/p with their surrounding cells that cover Ea/p upon gastrulation. I understand that those junctions move and may be at an awkward angle to image, so it may not be practical to quantify. In that case, please comment/clarify.

Related, the enrichment of afadin at the Ea/p junction appears to be consistent with notion of afadin/canoe responding to tension to secure adhesion (eg, Yu and Zallen, 2020). A brief discussion would help readers to make the connection and have a better understanding of what afadin may do in terms of coupling the mechanical forces.
We have now added this quantification as Fig 2F, and we have cited afadin's ability to localize "to sites under tension" (p. 12).

4. The Gad phenotype. The manuscript states the Gad phenotype as "the two EPCs failing to fully internalize or failing to stay internalized at the time the EPCs divided". These may be two different phenotypes with different interpretations of gene function. In particular, the latter may imply adhesion between the surrounding cells that cover Ea/p. For example, previous studies (eg, Pohl and Bao, 2012) showed dynamic interactions between the surrounding cells to seal over Ea/p. If the live imaging data allow, the authors should try to distinguish between the two phenotypes. If not, it'll be beneficial for the readers to provide a brief clarification/discussion.
We appreciate the reviewer's suggestion, although we have chosen to leave out this discussion. Our rationale is that Pohl et al's 2012 claim that MS progeny cells extend over EPCs is contentious; this is discussed with the relevant data presented in Goldstein and Nance 2020 (Genetics 214:265–277).

5. The transcriptome screen. The scRNA-seq dataset from the Murray and Waterston groups (Packer et al 2019) seems to provide better temporal and lineal resolution in terms of the gastrulating cells outside the E lineage. Could the authors comment on why they chose to conduct a marker-based sequencing?
We chose to commit the significant effort required – isolating cells expressing lineage markers from individual embryos of known stages – so that we could be confident of staging and cell identity, and to ensure that we had isolated fully intact cells (because unlysed cell fragments have been seen on occasion after manual shearing, suggesting that shearing can sometimes result in bisection of cells) and without risking reversals of incomplete cytokinesis by isolating cells before cytokinesis was complete (Edgar and Goldstein 2012). We now make this point in our Materials and Methods (p. 33).

6. The concept of slippage between actomyosin and cell boundary is central to the conclusions on afadin and zyxin function. It may be worthwhile to move the one-sentence definition of slippage from Methods to the main text to help the readers.
We provide this on p21, "This calculated difference in the movement of membrane and myosin is defined as the slippage rate".

Reviewer #3: The paper by Slabodnick, Tintori et. al. attempts to shed light on the mechanism by which the contracting apical actomyosin network is coupled to cell-cell junctions in order to facilitate apical constriction and cell invagination, using C. elegans gastrulation as a model. The paper sets out to find new proteins that could either serve as physical linkers themselves or regulate the timing of engagement of such linkers, both in the endodermal precursor cells and

more widely, in other gastrulating cells. The paper describes a mass spec experiment to identify proteins co-immunoprecipitating with HMP-1/α-catenin, data mining of published RNA-seq experiments and new single-cell RNAseq experiments to identify transcripts enriched specifically in ingressing cells just prior to their ingression. Based on these, the authors choose 32 candidate genes with which they perform an RNAi by injection screen for the Gad phenotype in EPCs. Of the resulting hits, they choose to follow up with some experiments that address the involvement of AFD-1 and ZYX-1 in actomyosin-junction coupling. The paper shows that systemic RNAi of afd-1 results in 25% gastrulation defects of Ea/Ep, that AFD-1 co-localizes with the cadherin/catenin complex, and that its junctional localization is cadherin-dependent. The paper also shows that systemic RNAi of zyx-1 results in 25% gastrulation defects of Ea/Ep and a zyx-1 null mutant has 58.8% Gad phenotype. RNAseq showed that zyx-1 transcripts are enriched the E lineage, and a highly overexpressed ZYX-1 fluorescent fusion protein could be detected in small foci at the apical junction as well as in the cytoplasm, but fluorescence microscopy failed to show expression of endogenously tagged ZYX-1. The paper describes a new semi-automated image analysis pipeline to analyze centripetal myosin flows, membrane movement, and the correlation between them in order to calculate slippage during Ea/Ep apical constriction, and they use this tool to show that slippage reduction over time is attenuated in zyx-1 null and afd-1 RNAi embryos, suggesting a role for worm zyxin and afadin in linking cell-cell junctions with apical actomyosin during gastrulation.

The paper addresses an important question and pursues logical hypotheses. Experiments are well-controlled and the resulting data are properly analyzed and presented. However, the paper in its current form suffers from three major problems. The first, is that it includes experiments (primarily with negative results) that don't advance the aims of the paper and don't contribute to its conclusions. The second, is that the evidence for the conclusion that zyxin and afadin directly link junctions to actomyosin within ingressing cells is weak, and is mostly based on what is known about their function in other systems, which leads me to the third point. Third, in choosing to focus on zyx-1 and afd-1, which are well known cytoskeletal linkers shown to be involved in apical constriction in other systems, and by performing only a limited number of experiments with these proteins, the paper falls short of providing new information on the mechanism of actomyosin-junction coupling.
We think this summary and the view that follows it below are unfair, based on misunderstanding some major parts of the manuscript.

The reviewer's first concern about negative results builds from their mistaken conclusion (below) that "none of the HMP-1 co-IP proteins ended up being pursued on the basis of the co-IP alone", and "this co-IP was not useful". In fact, our co-IP results were <u>essential</u> to identifying a role for afadin, which was then the basis for half of our original manuscript. The second concern, that we cannot nail that zyxin and afadin *directly* link junctions to actomyosin is fair, but it is the reason we submitted this manuscript to a genetics journal rather than a cell biology journal: identifying the relevant protein-encoding genes (a genetic problem) is the key issue faced in the work, because it is the key, current question based on the state of the field. The third concern about not providing new information on the mechanism of actomyosin-junction coupling – mirrored

below in their statement that afadin was known to link actomyosin and junctions in another system – misses a central point that is prominent in the Introduction: many such proteins are known, but none beyond cadherin (Roh-Johnson et al. 2012) were known to contribute to the dynamic linking of actomyosin and junctions that we study here. As we stated in our Introduction, "Although many proteins have been identified at sites of cadherin-based adhesion that could feasibly serve as such temporally regulated links, the specific, temporally-regulated links relevant to triggering apical constriction are not yet known in any system. Identifying such links is an important step toward understanding how developmental mechanisms orchestrate the cytoskeletal mechanisms of apical constriction with spatial and temporal precision." Importantly, the reviewer criticized the work for not examining actomyosin dynamics following *afd-1* or *zyx-1* depletion. This was in fact a central part of the manuscript.

In my view, in order for the paper to provide sufficient advance to the field to warrant publication in Plos Genetics it should either validate and substantiate the discovery of novel proteins that regulate actomyosin-junction coupling by performing protein localization and imaging of slippage for the other proteins in their list that gave a Gad phenotype, or provide novel mechanistic insight into the function of AFD-1 or ZYX-1 by performing, for example, cell-specific rescue experiments with various deletion constructs in the null background, or by performing more advanced imaging to visualize and quantify junctional and cytoskeletal protein dynamics under control and loss of function conditions.
We now present new cell-specific rescue results (p. 19), although as we note below, zyx-1 is only expressed in the endodermal founder cell. The new text on p. 19 reads, "In line with *zyx-1*'s expression enriched in only EPCs, P*sdz-1*-driven overexpression of full length mNG::ZYX-1a was able to rescue most of the defects seen in the *zyx-1Δ* background (2/27 Gad, *p* = 0.0003). Overexpression of mNG::LCR^{ZYX-1} was not able to rescue similarly (7/22 Gad, *p* = 0.366). We conclude that ZYX-1 contributes to gastrulation, and that domains beyond the LIM-domain-containing region are important for this function."

Specific comments:
1. In search of the link between junctions and apical actomyosin the authors screened for proteins from early-stage embryos that co-immunoprecipitate with α-catenin. This approach assumes that the connection with cadherin is mediated through α-catenin, which may be the case, but is not by any means the only way to connect actomyosin to the cell-cell junctions. The authors should state this limitation in their approach. Furthermore, in order to solubilize intact CCCs the lysis conditions must be fairly harsh, which means many weaker protein interactions would be lost. A BioID approach is probably more appropriate to answer this question.
We chose alpha-catenin for the pulldown because of our previous work implicating it as a relevant molecule (Roh-Johnson et al. 2012 *Science* 335:1232-5), and our finding that alpha-catenin is not pulled away from junctions by actomyosin contraction (Fig 1). Our approaches succeeded in identifying a protein that makes contributions to the coupling that we sought to understand. We agree that other approaches may identify additional players.

2. The authors narrow the list of candidates emerging from the Co-IP based on expression before or during the 24-cell stage of development using published single-cell mRNA sequencing data. Why don't they use the expression data they generated here for other lineages?
We did not use expression in other lineages because proteins that contribute to coupling need not be limited to specific cells. As we state (p. 25), gastrulation in *C. elegans* relies on embryonic transcription. But this does not imply that all relevant players must be regulated transcriptionally and in a lineage-specific manner.

3. AFD-1 and ZYX-1 were both candidates based on the EPC expression enrichment dataset. Hence, if I understood correctly, none of the HMP-1 co-IP proteins ended up being pursued on the basis of the co-IP alone. This makes me ask: if this co-IP was not useful why include it in the paper?
As we presented in the abstract and Results, this co-IP was indeed useful: AFD-1 was selected based on our co-IP result. *afd-1* mRNA was present at higher levels in stages before EPCs were born than in EPCs; we now make this clearer in Table 1 .

4. The results of the RNA-seq of internalizing cell types provides the community with new RNA expression data, but it does not, in the end, advance the goal of this paper. No "master regulator" of apical constriction was found and the idea that a family of LIM domain proteins, which are enriched in various gastrulating lineages, are common regulators of gastrulation is also not supported because RNAi of pxl-1 and lim-9 gave 0% gad. Therefore, in my opinion, this data unnecessarily complicates the paper and should be published separately.
The fact that no such regulator can be found despite this thorough, rigorous approach (using RNAseq of multiple lineages, at two timepoints) is a biological result that bears on the central questions we are asking in this manuscript. Therefore, we left the result in. We now articulate a goal of this effort more clearly when introducing this RNAseq approach, "finding that no such gene exists by exhaustive RNAseq analysis could also inform future models of apical constriction mechanisms by suggesting that apical constriction may be orchestrated by different regulators in different cells" (p. 13). We have also somewhat streamlined the writing in this section. We chose to submit to PLoS Genetics in part because publishing in PLoS Genetics permits us to present results concisely but fully, without length limits that could sacrifice key results like these.

5. Figure 5 and the results section describing the semi-automated workflow for analyzing actomyosin-membrane coupling, which is straight forward and based on established tools, belongs in the materials and methods section and not in the results section.
This is a new and potentially widely-applicable analysis pipeline requiring new code, and it is an essential step toward identifying genes that contribute to coupling. We acknowledge that coupling is a far more complex phenotype than assessed in typical screens, and we view this as a *strength* of the manuscript. Therefore, we have left this section in the manuscript but streamlined it to make it more concise.

6. The authors write that they follow up on zyx-1 and afd-1, which gave only 25% gad phenotype, because they are known junctional components. This is puzzling to me because in their RNAi screen several genes that are not known to regulate junction to medioapical actin connection had a higher parentage of gastrulation defects and would be, in my opinion, more interesting to study. For example, gei-4 had 42% Gad, hum-8 had 67%, and 46E10.8 had 50% Gad. As the authors note, afadin/canoe was already shown in Drosophila to be important for connecting medioapical actomyosin to cell-cell junctions, so showing it has a similar role in worms serves as a validation and it not novel.
Our approaches succeeded in identifying proteins that make contributions to the coupling that we sought to understand. We agree that other approaches, including work on other genes that we have implicated for the first time in gastrulation here, may identify additional players. The reviewer's comment about afadin's role in *Drosophila* misses a central point that is prominent in the Introduction: many proteins that can link actomyosin and junctions are known, but none beyond cadherin (Roh-Johnson et al. 2012) were known to contribute to the <u>dynamic</u> linking of actomyosin and junctions that we sought here.

7. A major caveat with all the RNAi experiments is that they are systemic and not cell specific. This is especially a problem with zyx-1 because it is expressed at very low levels in the Ea/Ep cells and therefore it raises the question whether the RNAi-induced phenotype isn't due to its depletion in other cells. EPC specific depletion of zyx-1 and afd-1 would be necessary to convincingly show that their expression in these cells is important for their invagination.
The reviewer has missed a central part of the manuscript: the RNAi phenotype of *zyx-1* cannot be due to depletion in other cells because *zyx-1* is only expressed in the E lineage (i.e., in EPCs; Fig 4C). We acknowledge that we do not know whether afd-1 functions within the EPCs, neighboring cells, or both, and our conclusions are in line with this.

8. Not being able to detect endogenous ZYX-1 in the EPCs is also a problem. It is possible to tag endogenous proteins with multiple fluorescent protein copies (e.g. 3XGFP) and this has previously been shown to allow the visualization of weakly expressed proteins. Visualizing endogenous zyxin would allow the authors to follow how its localization changes between early actomyosin contractions and coupled actomyosin contractions.
We have now tried using immunofluorescence to GFP to amplify a signal for ZYX-1::GFP in fixed embryos, and we have tried TSA amplification to try to boost signal; neither worked (p. 26). For live imaging, GFP is a poor choice for weakly expressed genes because 488nm illumination maximizes the otherwise low level background autofluorescence in *C. elegans* embryos; as we have described previously, mNG is the best fluorescent protein for this purpose (Heppert et al., 2016 *MBoC* 27:3385-94). It is possible that ZYX-1 functions specifically at very low levels as protein is first made, making it inherently difficult to visualize. We discuss this now on p. 17, "ZYX-1 might function specifically at a low level, during a time when levels are only beginning to rise transiently after initial gene expression."

9. An important question that was not addressed in the paper is how do CCC and actomyosin dynamics change following afd-1 or zyx-1 depletion. The authors used a membrane marker to image junctions and measure slippage, but it might be necessary to image components of the cadherin-catenin complex for this purpose.
A central point of our manuscript, highlighted by the title and abstract of the manuscript, is that we did in fact examine how actomyosin dynamics change following *afd-1* and *zyx-1* depletion (Figures 5 and 6). We agree that it will be interesting to determine also whether *afd-1* and *zyx-1* affect CCC dynamics, but our conclusions in the manuscript do not rely on this, and given reviewers' comments overall, we hesitate to add experiments that are not central to the main conclusions we are reaching.

10. The paper shows that AFD-1 co-localizes with HMR-1 and that it accumulates at the apical region during apical constriction. However, a more important question that should be addressed is how does its localization or amount change over time between high slippage and low slippage regimes?
AFD-1 localization does not change qualitatively over time; we have now added this as Fig 2D.

Minor comments:
1. In Table 1 the proteins are listed according to alphabetic order of gene name, which makes no sense. They should be divided according to the screen they came out of and ordered according to the severity of their gad phenotype.
We have now divided Table 1 (p. 11) according to the screen they came out of as suggested. We chose to not order genes by severity of phenotype because this was not the sole factor we used to pursue genes in the manuscript.

2. The authors write at the end of the first results section "these results imply that a strong connection between actomyosin and junctional α-catenin is initially missing in this system."- It is very likely that there are separate actomyosin networks at cell-cell junctions and in the apical region of the cell. Therefore, it should be stated more clearly that the authors conclude that a connection between the contracting apical actomyosin network and the CCC is initially missing, because there likely exists a connection between HMP-1 and junctional actomyosin at the same stage.
We appreciate the suggestion, and we have made the change (p. 8).

3. The authors found lower myosin and slippage rates overall than previously. It wasn't clear why this was the case and which numbers are more reflective of reality.
We did indeed find lower myosin and slippage rates overall than previously. As we stated in the manuscript (p. 21), this is not unexpected because our new workflow enables analysis of optical flow that considers smaller myosin particles than before, whose movements would be expected to be more affected by brownian motion.

4. Figure 3 should include images of the hmr-1 and sax-7 RNAi embryos
We have added these to Fig 3.

Suggestions for experiments that could add mechanistic insight:
1. The afd-1(RNAi) Gad phenotype is weak and the authors speculate that AFD-1 has a partially redundant role in C. elegans gastrulation. The authors could use an afd-1 mutant as a sensitized background to screen the other candidates by RNAi or do double RNAi with afd-1 plus each of the other candidates.
2. The authors speculate that AFD-1 might be post-translationally regulated differently in EPCs than in other cells. This could be tested with rescue experiments using mutants affecting specific PTMs.
We thank the reviewer for these suggestions. Given the revised manuscript's focus on ZYX-1, we have not added more data on AFD-1.
